# Supplementary material for: Structural basis for adhesion G protein-coupled receptor Gpr126 function
Source: Nat Commun. 2020 Jan 10;11:194. doi: 10.1038/s41467-019-14040-1 (PMC6954182; doi:10.1038/s41467-019-14040-1)
Supplement: Supplementary file 1 — Supplementary Information [file 41467_2019_14040_MOESM1_ESM.pdf]

Structural basis for adhesion G protein-coupled receptor Gpr126 function

Katherine Leon[1,2], Rebecca L. Cunningham[3], Joshua A. Riback[1,4], Ezra Feldman[1], Jingxian Li[1,2], Tobin R. Sosnick[1,5], Minglei Zhao[1], Kelly R. Monk[3,6], Demet Araç[1,2*]

[1]Department of Biochemistry and Molecular Biology, The University of Chicago, Chicago, IL, 60637, USA.

[2]Grossman Institute for Neuroscience, Quantitative Biology and Human Behavior, The University of Chicago, Chicago, IL, 60637, USA.

[3]Department of Developmental Biology, Washington University School of Medicine, St. Louis, MO, 63110, USA.

[4]Graduate Program in Biophysical Sciences Program, The University of Chicago, Chicago, IL, 60637, USA.

[5]Institute for Biophysical Dynamics, The University of Chicago, Chicago, IL, 60637, USA.

[6]Vollum Institute, Oregon Health & Science University, Portland, OR, 97239, USA.

*Corresponding author:
Demet Araç: arac@uchicago.edu

Supplementary Material:

Supplementary Figures 1-7

Supplementary Tables 1-2

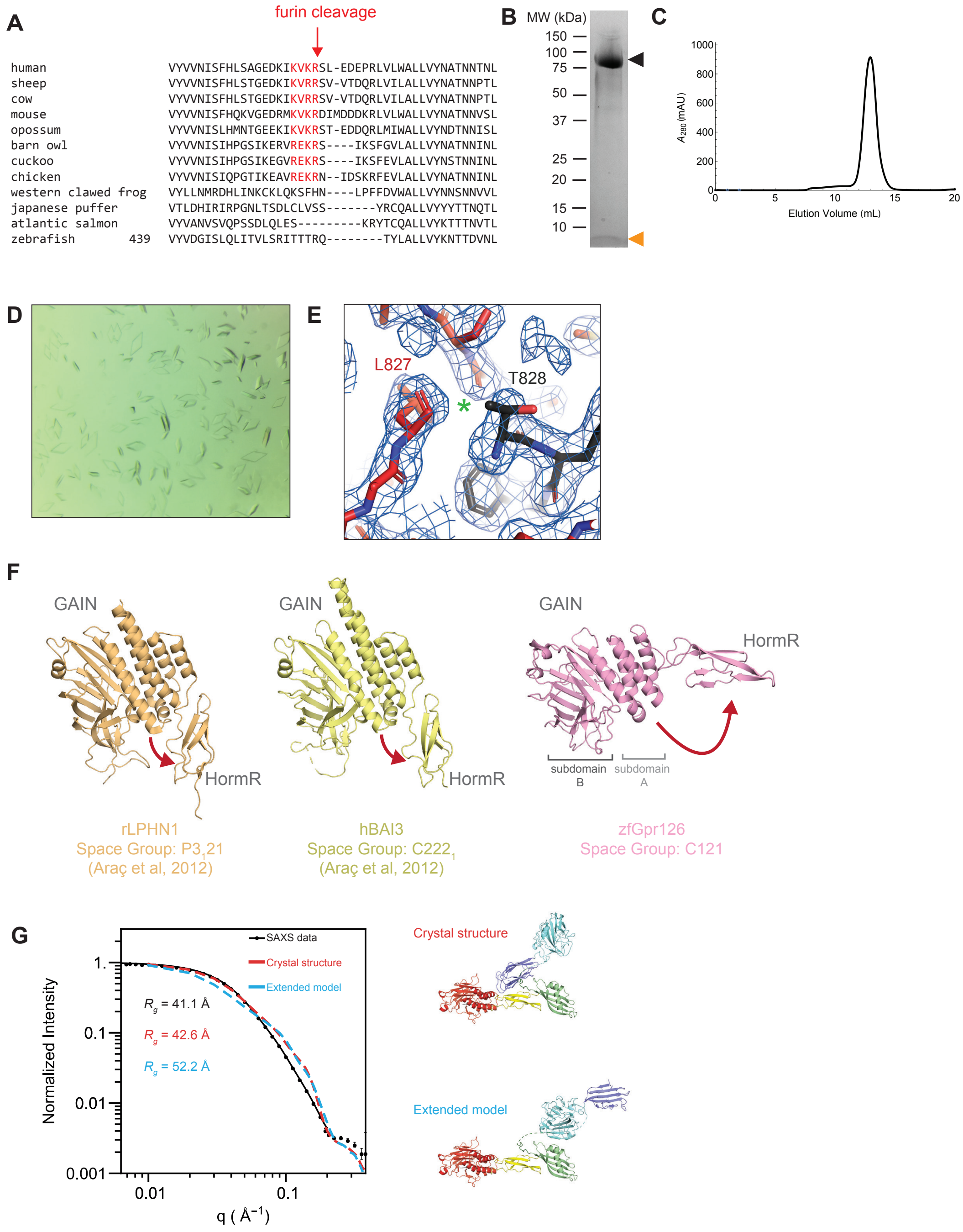

Supplementary Figure 1. Purification and crystallization of Gpr126 ECR. (A) Sequence alignment of Gpr126 furin-cleavage site from various species. Consensus cleavage site residues are colored red. (B) SDS-PAGE of purified Gpr126 ECR showing bands for N-terminal fragment (black arrowhead) and tethered peptide (orange arrowhead). Source data are provided as a Source Data file. (C) Size exclusion column profile of purified Gpr126 ECR. (D) Crystals of Gpr126 ECR. (E) 2Fo-Fc electron density map for Gpr126 ECR showing lack of electron density at autoproteolysis site (green asterisk) generating an N-terminal fragment (red) and C-terminal fragment (black). (F) HormR+GAIN domain structures and space groups of rLPHN1 (orange), hBAI3 (yellow), and zfGpr126 (pink). Red arrows indicate angles between GAIN and HormR domains, highlighting difference seen in zfGpr126 compared to all other structures. (G) Experimental SAXS curve for Gpr126 ECR (black) fit to a smooth regularized scattering curve (i.e. the Fourier transform of *p(r)*, solid black line, see methods), simulated SAXS curve for Gpr126 ECR based on crystal structure (red), and simulated SAXS curve for extended model of Gpr126 ECR (blue). Images of crystal structure and extended model used in the analysis are shown.

A B

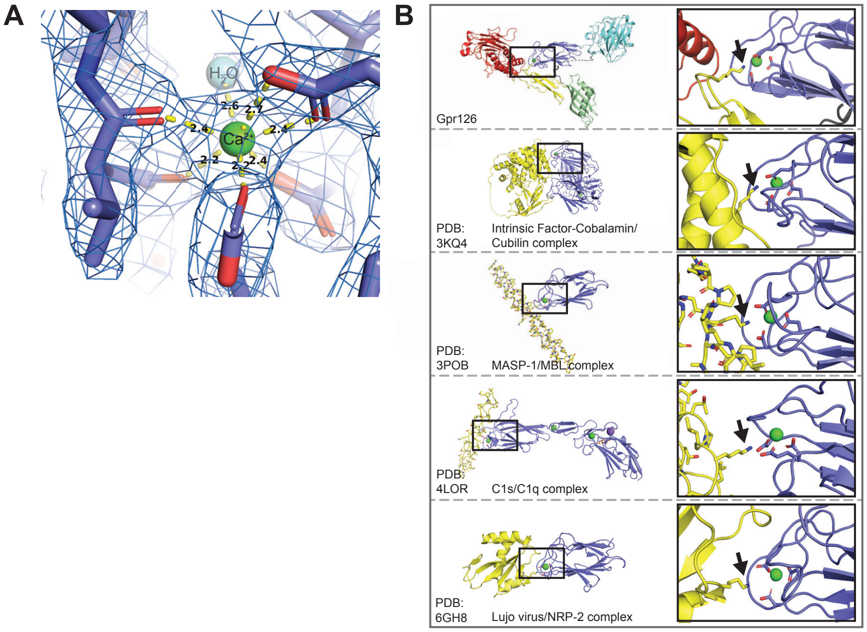

C

```
NRP2_CUB1          --CGGRLNSKDAGYITSPGYPQDYPSHQNCEWIVYAPEPNQKIVLNFNPHFEIE-----KHDCKYDFIEIRDGDSESADLLGKHCGNIAP----PTIISSGSMLYIKFTSDYARQG--AGFSLRYEIF
MASP1_CUB2         --CSGNLFTQRTGTITSPDYPNPYPKSSECSYTIDLEE-GFMVTLQFEDIFDIED--HPEVPCPYDYIKIKAGS----KVWGPFCGEKS----PEPISTQSHSIQILFRSDNSGEN--RGWRLSYRAA
C1s_CUB1           -------EPTMYGEILSPNYPQAYPSEVEKSWDIEVPE-GYGIHLYFT-HLDIEL----SENCAYDSVQIISGDTEEGRLCGQRSSNNPHSPIVEEFQVPYNKLQVIFKSDFSNEERFTGFAAYYVAT
MASP2_CUB1         TPLGPKWPEPVFGRLASPGFPGEYANDQERRWTLTAPP-GYRLRLYFT-HFDLEL----SHLCEYDFVKLSSGAKVLATLCGQESTDTERAPGKDTFYSLGSSLDITFRSDYSNEKPFTGFEAFYAAE
hGPR126_CUB        --CRVVLS-NPSGTFTSPCYPNDYPNSQACMWTLRAPT-GYIIQITFN-DFDIEE----APNCIYDSLSLDNGE-----SQTKFCGATAK---GLSFNSSANEMHVSFSSDFSIQK--KGFNASYIRV
zfGpr126_CUB   41  --CNVVLT-DSQGSFTSPCYPNDYPPSQSCNWTIQAPA-GFIVQITFL-DFELEE----AQGCIYDRVVVKTGT-----SDAKFCGLTAN---GLTLNSTGNVMEVFFNSDFSVQK--KGFHISYKQV
```

D

```
human                    ASCADLGTLCQ
sheep                    ASCADLGTLCQ
cow                      ASCADLGTLCQ
mouse                    TNCSELGTLCQ
opossum                  TSCADLGTLCQ
barn owl                 TSCANLGSLCQ
cuckoo                   TSCANLGSLCQ
chicken                  TSCANLGSLCQ
western clawed frog      TTCKNLGGVCQ
japanese puffer          TSCDPPGVGCP
atlantic salmon          TSCSSPGLDCP
zebrafish           367  AGCAS-GLGCP
                           └─S-S─┘
                        disulfide bond
```

Supplementary Figure 2. Analysis of the Gpr126 CUB domain. (A) 2Fo-Fc electron density map for Gpr126 ECR showing density for calcium ion (green sphere). Coordination interactions are shown as yellow dashed lines and distances (Å) are noted. (B) (Left panels) Structures of proteins that use calcium-binding CUB domains (blue) to bind ligands (yellow). (Right panels) Close-up views of the interfaces with arrows pointing to the lysine residues on the ligands which interact with calcium-binding residues on the CUB domains. (C) Sequence alignment of CUB domains from various proteins, highlighting conserved calcium-coordinating residues (red). (D) Alignment of the disulfide-bond loop region from various species showing the cysteines (bright orange) are conserved.

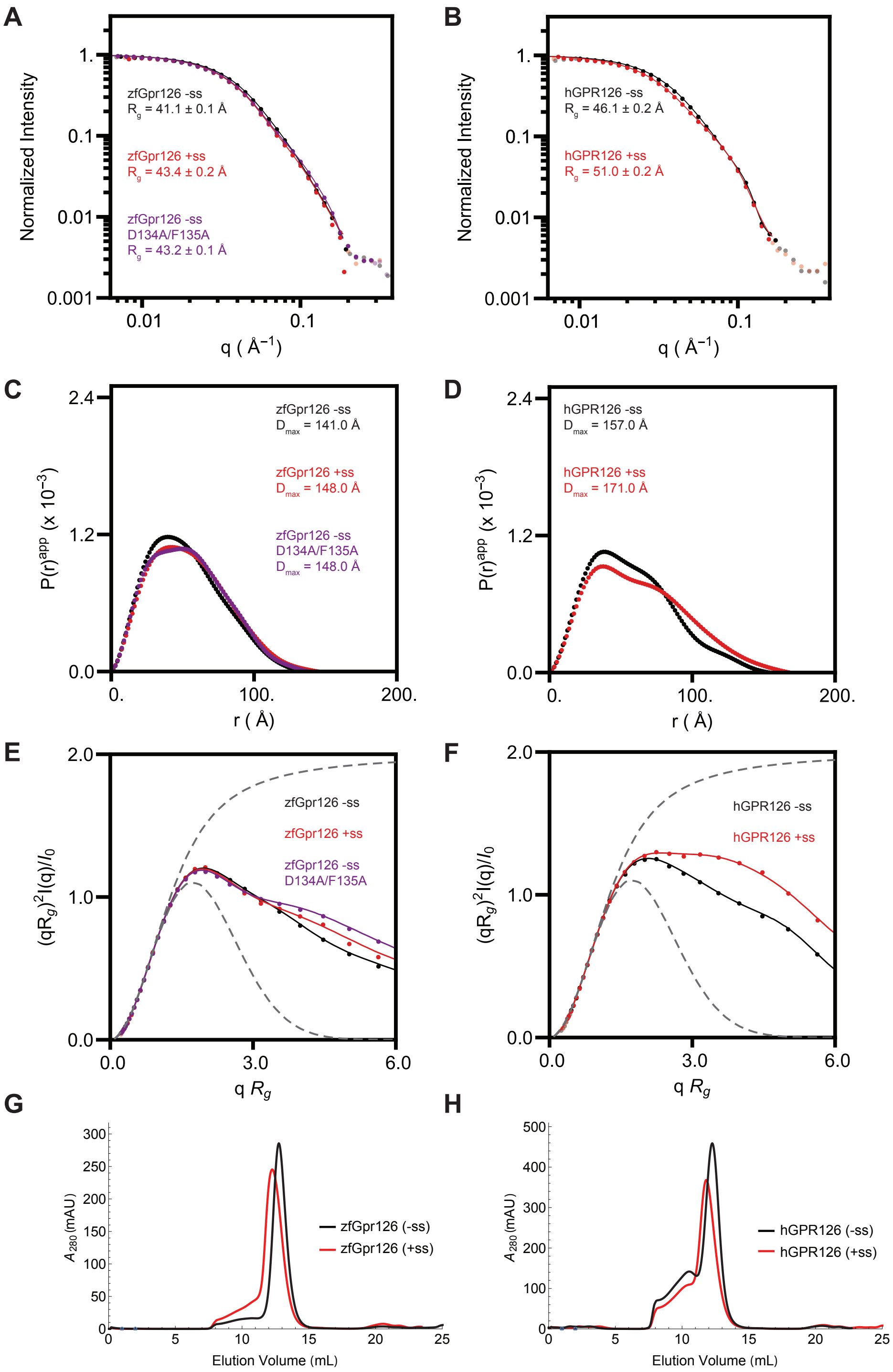

Supplementary Figure 3. Differences in ECR conformation between Gpr126/GPR126 splice isoforms. (A) SAXS curves for zebrafish Gpr126 (-ss) (black), (+ss) (red), and (-ss) D134A/F135A (purple). (B) SAXS curves for human R468A (furin-resistant) GPR126 (-ss) (black) and (+ss) (red). (C, D) *P(r)* plots for zebrafish Gpr126 (C) and human R468A GPR126 (D) constructs. $P(r)^{app}$ indicates a mixed protein/carbohydrate system (glycans present). (E, F) Normalized Kratky plots for zebrafish Gpr126 (E) and human R468A GPR126 (F) constructs. Dashed lines show profiles expected for a random walk and compact (Guinier) particle located at the top and bottom, respectively of the plots. Solid lines show experimental fit with a smooth regularized scattering curve (i.e. the Fourier transform of *p(r)*, solid black line, see methods). (G, H) Size exclusion column profiles for zebrafish Gpr126 and human R468A GPR126 splice isoforms.

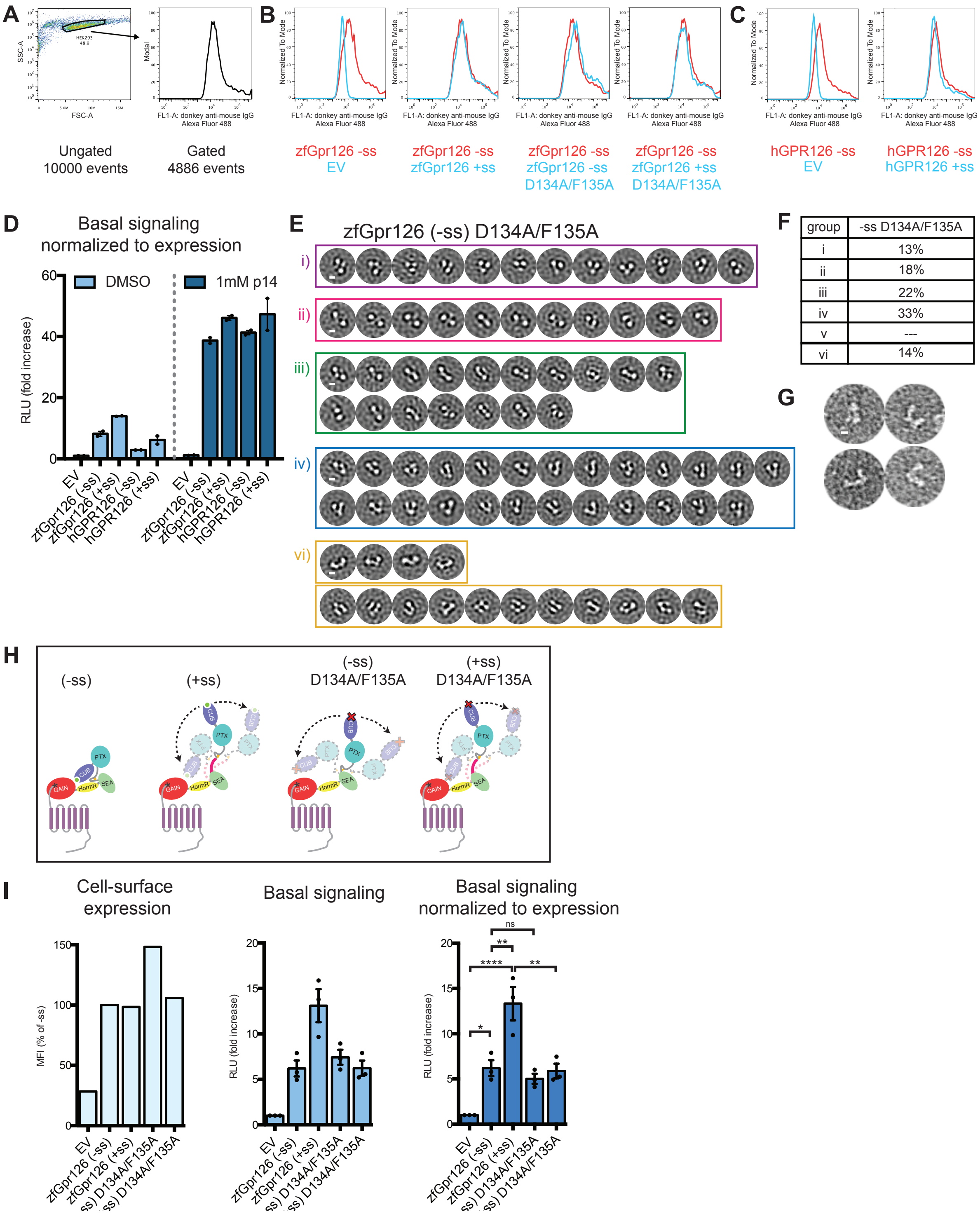
A
SSC-A
FSC-A
HEK293
48.9
Modal
FL1-A: donkey anti-mouse IgG Alexa Fluor 488
Ungated
10000 events
Gated
4886 events
B
Normalized To Mode
zfGpr126 -ss
EV
zfGpr126 -ss
zfGpr126 +ss
zfGpr126 -ss
zfGpr126 -ss D134A/F135A
zfGpr126 -ss
zfGpr126 +ss D134A/F135A
C
hGPR126 -ss
EV
hGPR126 -ss
hGPR126 +ss
D
Basal signaling normalized to expression
DMSO
1mM p14
RLU (fold increase)
EV
zfGpr126 (-ss)
zfGpr126 (+ss)
hGPR126 (-ss)
hGPR126 (+ss)
E
zfGpr126 (-ss) D134A/F135A
i)
ii)
iii)
iv)
vi)
F
group | -ss D134A/F135A
i | 13%
ii | 18%
iii | 22%
iv | 33%
v | ---
vi | 14%
G
H
(-ss)
(+ss)
(-ss) D134A/F135A
(+ss) D134A/F135A
PTX
CUB
GAIN
HormR
SEA
I
Cell-surface expression
MFI (% of -ss)
Basal signaling
RLU (fold increase)
Basal signaling normalized to expression
ns
*
**
****
EV
zfGpr126 (-ss)
zfGpr126 (+ss)
ss) D134A/F135A

Supplementary Figure 4. Analysis of Gpr126 cAMP signaling and ECR conformation. (A) Representative gating strategy to sort HEK293 cells for cultures presented on Supplementary Figures 4B, C, I and Figures 4A, D, based on forward scatter (FSC) and side scatter (SSC). Gated cells are shown in a green fluorescence (FL1) histogram. (B, C) Surface expression of zebrafish Gpr126 (B) and human GPR126 (C) constructs presented as overlay of typical flow cytometry histograms. HEK293 cells transfected with N-terminal FLAG-tagged full-length Gpr126/GPR126 constructs or empty vector (EV) were stained with anti-FLAG antibody without permeabilization. Expression (i.e. FLAG signal) for cells transfected with each construct (blue) is normalized to expression for cells transfected with the zebrafish or human -ss isoform (red). (D) Basal (left) and activated (right) cAMP signaling normalized to cell-surface expression for empty vector (EV), zebrafish Gpr126 splice isoforms, and human GPR126 splice isoforms. Data presented as mean ± SEM, n = 2. (E) Negative stain EM 2D class averages for zebrafish Gpr126 -ss D134A/F135A ECR constructs. Class averages are categorized according to similar orientations as in Figure 3. (i, ii, iii, iv) are observed in both -ss and +ss isoforms as well as the -ss D134A/F135A mutant. (vi) represents open-like conformations. Scale bars (white) represent 50 Å. (F) Quantification of percentage of particles per category. (G) Representative individual particles for -ss D134A/F135A. (H) ECR conformations are depicted as cartoons. The red cross represents the D134A/F135A mutation. Black arrows with dashed lines indicate dynamic ECR conformation. Autoproteolysis in GAIN domain is indicated by a black asterisk. (I) (left) Cell-surface expression levels for EV, zebrafish Gpr126 splice isoforms, and mutant D134A/F135A isoforms, measured using flow cytometry to detect binding of anti-FLAG antibody to cells expressing FLAG-tagged Gpr126. The Gpr126 cell-surface expression levels are normalized to the control EV signal. Error bars are not shown because expression levels are presented as median fluorescence intensities of 10,000 cells for each population of transfected cells, for a single flow cytometry experiment representative of at least three independent experiments. (center) Basal signaling measured by the cAMP signaling assay. Data are shown as fold increase over EV of RLU (relative luminescence units). (right) Basal cAMP signaling normalized to cell-surface expression. ns, $P > 0.05$; *, $P \leq 0.05$; **, $P \leq 0.01$; ***, $P \leq 0.001$; ****, $P \leq 0.0001$; by one-way ANOVA and Tukey's multiple comparisons test. Signaling data are presented as mean ± SEM, n = 3, and are representative of at least three independent experiments. Source data are provided as a Source Data file.

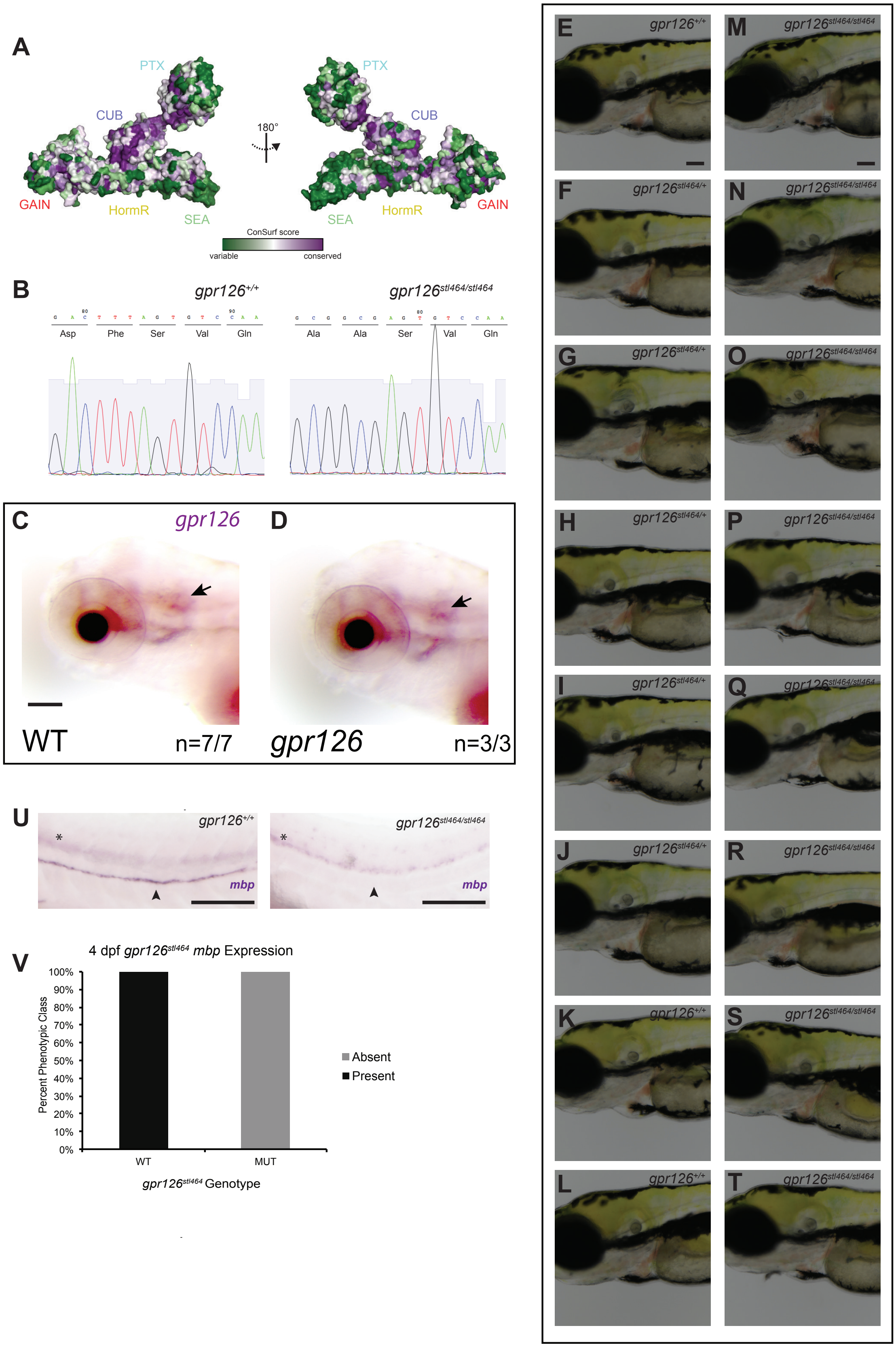
A
PTX
CUB
180°
GAIN
HormR
SEA
ConSurf score
variable
conserved
B
gpr126+/+
gpr126stl464/stl464
Asp
Phe
Ser
Val
Gln
Ala
Ala
Ser
Val
Gln
C
gpr126
D
WT
n=7/7
gpr126
n=3/3
U
gpr126+/+
gpr126stl464/stl464
mbp
mbp
V
4 dpf gpr126stl464 mbp Expression
Percent Phenotypic Class
100%
90%
80%
70%
60%
50%
40%
30%
20%
10%
0%
WT
MUT
gpr126stl464 Genotype
Absent
Present
E gpr126+/+
F gpr126stl464/+
G gpr126stl464/+
H gpr126stl464/+
I gpr126stl464/+
J gpr126stl464/+
K gpr126+/+
L gpr126+/+
M gpr126stl464/stl464
N gpr126stl464/stl464
O gpr126stl464/stl464
P gpr126stl464/stl464
Q gpr126stl464/stl464
R gpr126stl464/stl464
S gpr126stl464/stl464
T gpr126stl464/stl464

Supplementary Figure 5. Mutation of a conserved site in the ECR of Gpr126 is abolishes myelination. (A) Conservation score (green, variable; purple, conserved) of each residue mapped onto the surface of Gpr126 ECR. (B) Sequence traces for *gpr126*$^{+/+}$ *and gpr126*$^{stl464/stl464}$ zebrafish, showing D134A/F135A mutations in *gpr126*$^{stl464/stl464}$. (C, D). *In situ* hybridization shows similar expression of *gpr126* in 5 dpf wild-type siblings (C) and *gpr126*^{stl464} mutants (D). Arrows denote ears. Scale bar (black) represents 100 µm. (E-L) 4 dpf wild-type and *gpr126*$^{464/+}$ larvae. (M-T) 4 dpf *gpr126*$^{464/464}$ larvae. No substantial heart defects were observed in mutants at this resolution. Scale bars (black) represent 100 µm. (U) 4 dpf wild-type larvae express *mbp* throughout the posterior lateral line nerve (PLLn, arrowhead), whereas 4 dpf *gpr126*$^{stl464/stl464}$ larva lack *mbp* expression along the PLLn (arrowhead). Scale bars (black) represent 100 µm. Asterisks indicate CNS. (V) Quantification of presence or absence of *mbp* expression in wild-type and mutant larvae.

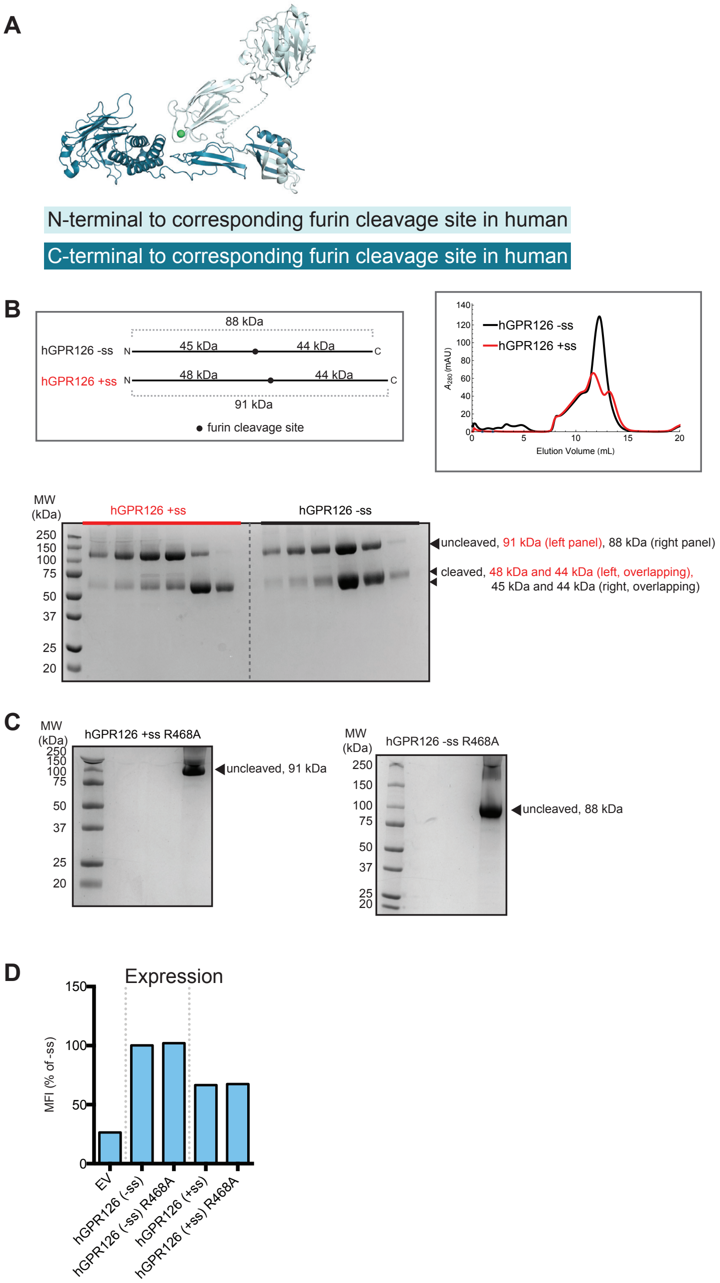

Supplementary Figure 6. Furin cleavage of GPR126. (A) Structure of Gpr126 ECR. The region N-terminal to the corresponding human furin cleavage site is colored light blue and the region C-terminal to the corresponding human furin cleavage site is colored dark blue. (B) (top, left) Molecular weights for full-length ECR and furin-cleaved ECR fragments of human GPR126 -ss and +ss isoforms are shown, with the furin cleavage site shown as a black circle. (top, right) Size exclusion column profiles for human GPR126 -ss (black) and +ss (red) ECR proteins. (bottom) SDS-PAGE analysis of size exclusion column fractions, showing partial cleavage by furin of both isoforms. The top bands (~88, 91 kDa) correspond to uncleaved GPR126 -ss and +ss ECR, respectively. The bottom bands (~45/44 and 48/44 kDa) correspond to cleaved GPR126 -ss and +ss ECR, respectively. The N-terminal and C-terminal cleaved fragments are close in size and overlap on the gel. (C) SDS-PAGE analysis of human GPR126 -ss (left) and +ss (right) ECR constructs with furin-resistant R468A mutations, showing loss of cleaved fragments. Source data are provided as a Source Data file. (D) GPR126 cell-surface expression levels on HEK293 cells transfected with empty vector (EV) and various human GPR126 constructs. Error bars are not shown because expression levels are presented as median fluorescence intensities of 10,000 cells for each population of transfected cells, for a single flow cytometry experiment representative of at least three independent experiments.

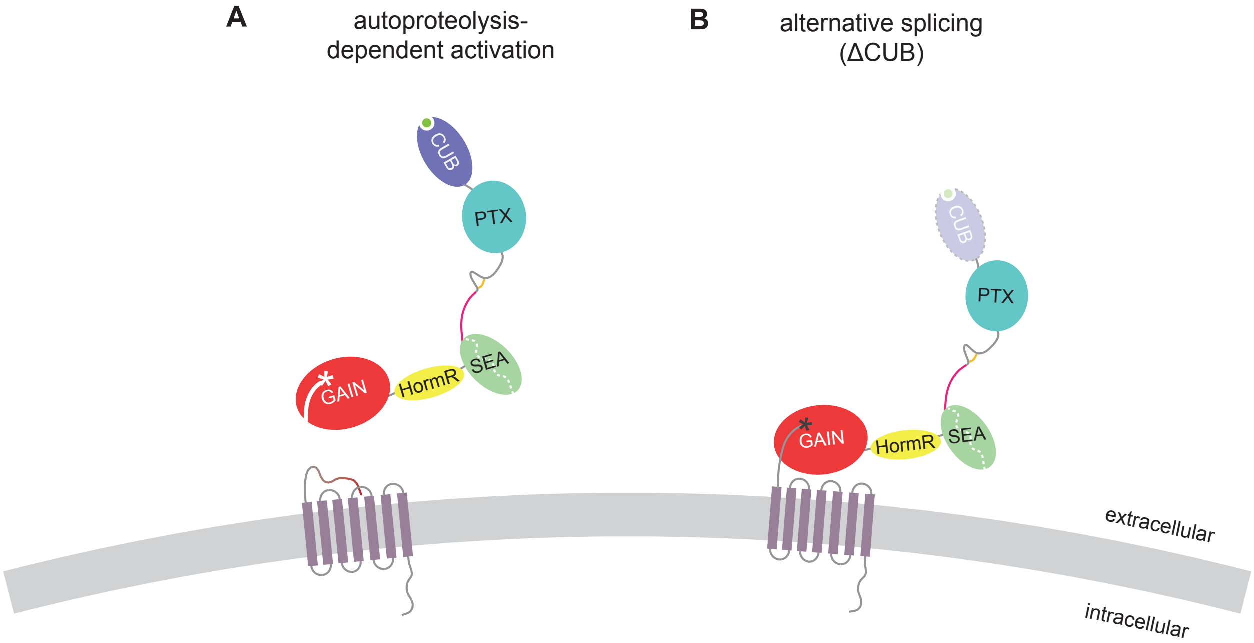

Supplementary Figure 7. Additional models for activation and regulation of Gpr126 function. (A) Mechanical force exerted on the Gpr126 ECR leads to exposure of tethered peptide and activation of the receptor. (B) An alternative splice isoform lacks the CUB domain and may be a mechanism for receptor regulation. Autoproteolysis in GAIN domain is indicated by an asterisk. The calcium ion is shown as a bright green circle.

Supplementary Table 1. Data Collection and Refinement Statistics

Protein	Gpr126 ECR Native Dataset	Gpr126 ECR SeMet SAD
Data Collection		
Integration Package	HKL2000	HKL2000
Wavelength	1.033 Å	0.979 Å
Space group	C2	C2
Cell dimensions		
a, *b*, *c*	144.96 Å, 59.45 Å, 168.39 Å	145.20 Å, 59.33 Å, 168.72 Å
α, β, γ	90°, 107.82°, 90°	90°, 108.16°, 90°
Resolution	50–2.40 Å (2.46–2.40 Å)	50–2.34 Å (2.38–2.34 Å)
R_{sym} or R_{merge}	0.064 (0.664)	0.086 (0.492)
$CC_{1/2}$, highest res. bin	0.557	0.376
$I/\sigma I$	15.0 (0.9)	13.0 (1.4)
Completeness	77.9% (25.4%)	71.6% (9.7%)
Redundancy	2.9 (1.7)	3.8 (1.3)
Number of measured reflections	125687	157602
Number of unique reflections	43044	41385
Refinement Statistics		
R_{work}/R_{free}	0.2121/0.2717	
Number of atoms		
Protein	6114	
Water	197	
Other	155	
Average B-factors		
Protein	39.0 $Å^2$	
Water	30.42 $Å^2$	
Other	61.67 $Å^2$	
Rmsds		
Bond lengths	0.010 Å	
Bond angles	1.21 °	
Ramachandran plot statistics		
Most favorable	95.03%	
Allowed	4.83%	
Disallowed	0.13%	

Supplementary Table 2. SAXS analysis for Gpr126/GPR126 constructs

(*a*) Sample details

	zfGpr126 -ss ECR	zfGpr126 +ss ECR	zfGpr126 -ss ECR D134A/F135A	hGPR126 -ss ECR R468A	hGPR126 +ss ECR R468A
Organism	Zebrafish	Zebrafish	Zebrafish	Human	Human
Source (Catalogue No. or reference)	Monk Lab	Monk Lab	Monk Lab	Addgene plasmid #66314	Addgene plasmid #66314
Description	C6KFA3 splice variant S2, C-terminal 8xHis tag	C6KFA3 splice variant S1, C-terminal 8xHis tag	C6KFA3 splice variant S2 with D134A and F135A mutations, C-terminal 8xHis tag	Q86SQ4, C-terminal 8xHis tag, R468A mutation	Q86SQ4, C-terminal 8xHis tag, R468A mutation
Extinction coefficient ε (A280, 0.1%(w/v))	1.094	1.066	1.096	1.195	1.155
Partial specific volume $\bar{\bar{\nu}}$ (cm^3 g^{-1})	0.733	0.734	0.733	0.735	0.734
Mean solute and solvent scattering length densities and mean scattering contrast $\Delta\bar{\bar{\rho}}$ (10^{10} cm^{-2})	2.892 (12.352 – 9.460)	2.886 (12.346 – 9.460)	2.891 (12.351 – 9.460)	2.874 (12.334 – 9.460)	2.881 (12.341 – 9.460)
Molecular mass *M* from chemical composition (Da)	86371	88708	86251	88444	91467
SEC-SAXS column	Superdex 200 10/300				
Loading concentration, (mg ml^{-1})	5.0	2.7	2.5	4.2	4.2
Injection volume (µl)	200	200	200	200	200
Flow rate (ml min^{-1})	0.7	0.7	0.7	0.7	0.7
Solvent composition and source	20 mM HEPES pH 7.2, 150 mM NaCl				

(*b*) SAS data collection parameters

Source, instrument and description or reference	Advanced Photon Souce at Argonne National Labs, BioCAT 18-ID with Pilatus 3 1M detector
Wavelength (Å)	1.03
Beam size (µm)	172 x 172
Sample-to-detector distance (m)	1.0
q-measurement range ($Å^{-1}$)	0.00540153 – 0.383203
Absolute scaling method	N/A
Basis for normalization to constant counts	Incident flux

Method for monitoring radiation damage, X-ray dose where relevant	Dose < 10^{12} photons sec^{-1}.
Exposure time, number of exposures	Continuous 0.8 s data-frame measurements of SEC elution
Sample configuration including path length and flow rate where relevant	In-line SEC-SAXS, pathlength = 1.5 mm at 0.7 ml min^{-1}.
Sample temperature (ºC)	22

(*c*) Software employed for SAS data reduction, analysis and interpretation

SAS data reduction to sample–solvent scattering, and extrapolation, merging, desmearing *etc*. as relevant	BioXTAS RAW[1]
Calculation of ε from sequence	ProtParam[2]
Calculation of $\bar{\bar{v}}$ and $\Delta\bar{\rho}$ values from chemical composition	MULCh[3]
Basic analyses: Guinier, *P*(*r*), scattering particle volume (*e.g.* Porod volume V_P or volume of correlation V_c)	BioXTAS RAW[1]<br>AUTORG, DATGNOM from ATSAS[4]
Shape/bead modelling	N/A
Atomic structure modelling (homology, rigid body, ensemble)	CRYSOL[5]
Modelling of missing sequence from PDB files	N/A
Molecular graphics	The PyMOL Molecular Graphics System, Version 2.0 Schrödinger, LLC

(*c*) Structural parameters

	zfGpr126 -ss ECR	zfGpr126 +ss ECR	zfGpr126 -ss ECR D134A/F135A	hGPR126 -ss ECR R468A	hGPR126 +ss ECR R468A
Guinier Analysis					
I(0)	395.1964 ± 0.8711	90.2641 ± 0.4052	153.6211 ± 0.4943	124.1835 ± 0.4630	129.0501 ± 0.5463
R_g (Å)	40.1790 ± 0.1624	42.4661 ± 0.2703	42.8000 ± 0.2312	44.0908 ± 0.2382	48.9428 ± 0.2815
q-range ($Å^{-1}$)	0.0054 – 0.02957	0.00642 – 0.03119	0.00642 – 0.02843	0.00642 – 0.02935	0.0052 – 0.02721
Coefficient of correlation, R^2	0.9937	0.9840	0.9902	0.9704	0.9737
P(*r*) analysis					
I(0)	398.100 ± 0.6880	90.7600 ± 0.3192	153.9000 ± 0.4063	126.1000 ± 0.3874	130.7000 ± 0.4821
R_g (Å)	41.130 ± 0.098	43.400 ± 0.189	43.240 ± 0.144	46.070 ± 0.193	50.980 ± 0.225
d_{max} (Å)	141	148	148	157	171
q-range ($Å^{-1}$)	0.0076 – 0.1991	0.0087 – 0.1890	0.0052 – 0.3663	0.0102 – 0.1777	0.0076 – 0.1638
Total estimate from GNOM	0.915	0.825	0.9051	0.804	0.755
Porod volume ($Å^{-3}$)	169000	191000	181000	199000	213000

(*d*) Deposition IDs

	zfGpr126 -ss ECR	zfGpr126 +ss ECR	zfGpr126 -ss ECR D134A/F135A	hGPR126 -ss ECR R468A	hGPR126 +ss ECR R468A
SASBDB ID	SASDFT9	SASDFU9	SASDFV9	SASDFW9	SASDFX9

Supplementary References

1. Hopkins JB, Gillilan RE, Skou S. BioXTAS RAW: improvements to a free open-source program for small-angle X-ray scattering data reduction and analysis. *Journal of applied crystallography* **50**, 1545-1553 (2017).

2. Gasteiger E*, et al.* Protein Identification and Analysis Tools on the ExPASy Server. In: *The Proteomics Protocols Handbook* (ed^(eds Walker JM). Humana Press (2005).

3. Whitten AE, Cai S, Trewhella J. MULCh: modules for the analysis of small-angle neutron contrast variation data from biomolecular assemblies. *Journal of applied crystallography* **41**, 222-226 (2008).

4. Petoukhov MV, Konarev PV, Kikhney AG, Svergun DI. ATSAS 2.1 - towards automated and web-supported small-angle scattering data analysis. *Journal of applied crystallography* **40**, s223-s228 (2007).

5. Petoukhov MV*, et al.* New developments in the ATSAS program package for small-angle scattering data analysis. *Journal of applied crystallography* **45**, 342-350 (2012).
