# Supplementary material for: Structural basis for adhesion G protein-coupled receptor Gpr126 function
Source: Nat Commun. 2020 Jan 10;11:194. doi: 10.1038/s41467-019-14040-1 (PMC6954182; doi:10.1038/s41467-019-14040-1)
Supplement: Supplementary file 3 — Description of Additional Supplementary Files [file 41467_2019_14040_MOESM3_ESM.docx]

**Description of Supplementary Files**

**File Name:** Supplementary Data 1

**Description:** Alternative splicing, furin cleavage, and disulfide loop analysis for various Gpr126 species.
