# Supplementary material for: Structural basis for adhesion G protein-coupled receptor Gpr126 function
Source: Nat Commun. 2020 Jan 10;11:194. doi: 10.1038/s41467-019-14040-1 (PMC6954182; doi:10.1038/s41467-019-14040-1)
Supplement: Supplementary file 5 — Reporting Summary [file 41467_2019_14040_MOESM5_ESM.pdf]

nature research

Corresponding author(s): Demet Araç

Last updated by author(s): Oct 30, 2019

Reporting Summary

Nature Research wishes to improve the reproducibility of the work that we publish. This form provides structure for consistency and transparency in reporting. For further information on Nature Research policies, see Authors & Referees and the Editorial Policy Checklist .

Statistics

For all statistical analyses, confirm that the following items are present in the figure legend, table legend, main text, or Methods section.

n/a	Confirmed	
☐	☒	The exact sample size (*n*) for each experimental group/condition, given as a discrete number and unit of measurement
☐	☒	A statement on whether measurements were taken from distinct samples or whether the same sample was measured repeatedly
☐	☒	The statistical test(s) used AND whether they are one- or two-sided<br>*Only common tests should be described solely by name; describe more complex techniques in the Methods section.*
☒	☐	A description of all covariates tested
☒	☐	A description of any assumptions or corrections, such as tests of normality and adjustment for multiple comparisons
☐	☒	A full description of the statistical parameters including central tendency (e.g. means) or other basic estimates (e.g. regression coefficient) AND variation (e.g. standard deviation) or associated estimates of uncertainty (e.g. confidence intervals)
☒	☐	For null hypothesis testing, the test statistic (e.g. *F*, *t*, *r*) with confidence intervals, effect sizes, degrees of freedom and *P* value noted<br>*Give P values as exact values whenever suitable.*
☒	☐	For Bayesian analysis, information on the choice of priors and Markov chain Monte Carlo settings
☒	☐	For hierarchical and complex designs, identification of the appropriate level for tests and full reporting of outcomes
☒	☐	Estimates of effect sizes (e.g. Cohen's *d*, Pearson's *r*), indicating how they were calculated

Our web collection on statistics for biologists contains articles on many of the points above.

Software and code

Policy information about availability of computer code

Data collection	JBluIce-EPICS (x-ray diffraction collection), BioTek Gen5 (signaling data collection), FEI Tecnai (electron microscopy collection)
Data analysis	HKL2000 (x-ray diffraction processing), PHENIX (x-ray structure determination), CCP4 (x-ray structure determination), BioXTAS RAW (SAXS processing), GraphPad Prism (signaling data analysis), PyMOL (structure visualization), EMAN2 (electron microscopy analysis), ConSurf (structural conservation analysis), Dali server (structural comparison)

For manuscripts utilizing custom algorithms or software that are central to the research but not yet described in published literature, software must be made available to editors/reviewers. We strongly encourage code deposition in a community repository (e.g. GitHub). See the Nature Research guidelines for submitting code & software for further information.

Data

Policy information about availability of data

All manuscripts must include a data availability statement. This statement should provide the following information, where applicable:
- Accession codes, unique identifiers, or web links for publicly available datasets
- A list of figures that have associated raw data
- A description of any restrictions on data availability

The accession number for the coordinates and diffraction data for the Gpr126 (-ss) ECR crystal structure reported in this paper is PDB: XXXX. The SASBDB IDs for the SAXS experimental data are: SASDFT9, SASDFU9, SDSDFV9, SASDFW9, SASDFX9.

Field-specific reporting

Please select the one below that is the best fit for your research. If you are not sure, read the appropriate sections before making your selection.

Life sciences study design

All studies must disclose on these points even when the disclosure is negative.

Sample size	Three samples of each transfected cell population were used in signaling assays. Twenty zebrafish of each background were evaluated in animal studies. Sample sizes are consistent with similar previously published studies.
Data exclusions	No data were excluded.
Replication	Signaling assays were done in triplicate, and all attempts at replication were successful.
Randomization	Cells were placed in different positions on 96-well signaling assay plate.
Blinding	Blinding is not relevant to this study due to the all-or-none zebrafish phenotypes.

Reporting for specific materials, systems and methods

We require information from authors about some types of materials, experimental systems and methods used in many studies. Here, indicate whether each material, system or method listed is relevant to your study. If you are not sure if a list item applies to your research, read the appropriate section before selecting a response.

Materials & experimental systems

n/a	Involved in the study	
☐	☒	Antibodies
☐	☒	Eukaryotic cell lines
☒	☐	Palaeontology
☐	☒	Animals and other organisms
☒	☐	Human research participants
☒	☐	Clinical data

Methods

n/a	Involved in the study	
☒	☐	ChIP-seq
☐	☒	Flow cytometry
☒	☐	MRI-based neuroimaging

Antibodies

Antibodies used	mouse anti-FLAG (Sigma-Aldrich, F3165), donkey anti-mouse Alexa Fluor 488 (Invitrogen, A21202)
Validation	Most commercially available antibodies (from various suppliers) that are being used during the course of our studies have been validated by our lab and others through comparison of obtained immunohistochemistry (IHC) and/or Western blot (WB) results between labs and, in many cases, confirmation of specificity using mutant mice lacking the proteins detected by the antibodies. We validate each new lot of antibody by reproducing results obtained with previous batches of antibody.<br><br>(from Sigma-Aldrich website) Anti Flag M2 antibody is used for the detection of Flag fusion proteins.This monoclonal antibody is produced in mouse and recognizes the FLAG sequence at the N-terminus, Met N-terminus, and C-terminus. The antibody is also able to recognize FLAG at an internal site. M2, unlike M1 antibody is not Calcium dependent. Antibody is recommended for use in immunoblotting, immunoprecipitation, immunocytochemistry, immunofluorescence, ELISA, electron microscopy, flow cytometry and supershift assays.

Eukaryotic cell lines

Policy information about cell lines

Cell line source(s)	ATCC: HEK293<br>Invitrogen: SF9, High Five
Authentication	HEK293 cell lines (from ATCC) have been validated by the supplier through STR analysis and cytogenetic studies. We also employ a protocol for sequencing of baculoviral stocks postamplification to confirm identities of proteins over-expressed in lepidopteran insect cells, especially for mutant variants that are hard to differentiate otherwise.
Mycoplasma contamination	Cell line tested negative for mycoplasma contamination (ATCC)
Commonly misidentified lines<br>(See ICLAC register)	N/A

Animals and other organisms

Policy information about studies involving animals; ARRIVE guidelines recommended for reporting animal research

Laboratory animals	Zebrafish were maintained in the Washington University Zebrafish Consortium Facility (http://zebrafish.wustl.edu), and the following experiments were performed according to Washington University animal protocols. The gpr126stl464 zebrafish were generated within the wild-type AB* background. All crosses were either set up as pairs or harems and embryos were raised at 28.5° C in egg water (5 mM NaCl, 0.17 mM KCl, 0.33 mM CaCl2, 0.33 mM MgSO4). Larvae were staged at days post fertilization (dpf).
Wild animals	N/A
Field-collected samples	N/A
Ethics oversight	Washington University Zebrafish Consortium Facility

Note that full information on the approval of the study protocol must also be provided in the manuscript.

Flow Cytometry

Plots

Confirm that:

- [x] The axis labels state the marker and fluorochrome used (e.g. CD4-FITC).
- [x] The axis scales are clearly visible. Include numbers along axes only for bottom left plot of group (a 'group' is an analysis of identical markers).
- [x] All plots are contour plots with outliers or pseudocolor plots.
- [x] A numerical value for number of cells or percentage (with statistics) is provided.

Methodology

Sample preparation	Samples are transiently transfected HEK293 cells. Flow cytometry was used to measure cell surface expression of transiently expressed proteins.
Instrument	BD Accuri C6
Software	FlowJo
Cell population abundance	No sorting was performed.
Gating strategy	Cell populations were gated only in forward scatter vs side scatter plot to separate cells from debris.

- [x] Tick this box to confirm that a figure exemplifying the gating strategy is provided in the Supplementary Information.
